# The need for new "patient-related" guidelines for the treatment of acute cholecystitis

**DOI:** 10.1186/1749-7922-6-44

**Published:** 2011-12-22

**Authors:** Fabio C Campanile, Fausto Catena, Federico Coccolini, Marco Lotti, Dario Piazzalunga, Michele Pisano, Luca Ansaloni

**Affiliations:** 1 Department of Surgery, ASL VT-San Giovanni Decollato-Andosilla Hospital, via Ferretti 169 Civita Castellana 01033, Italy; 2 Department of General and Transplant Surgery, Sant'Orsola-Malpighi Hospital, Bologna, Italy; 3 Department of General and Emergency Surgery, Ospedali Riuniti, Bergamo, Italy

## Abstract

Heterogeneity of patients affected by acute cholecystitis, and their co-morbidities make very difficult to standardize the therapy for this very common condition. The staging system suggested in the recent "Tokyo guidelines", did not show a relevant impact on the management of patients and on the outcome of the disease. The relation among local pathological picture, patient clinical status and treatment algorithm, has to be better studied.

## Editorial

As universally known, acute cholecystitis is a frequent complication of cholelithiasis. It is a very common problem and general surgeons have to face it daily. The absolute heterogeneity of patients, co-morbidities and environment in which this disease presents, make the diagnosis, and the subsequent therapeutic procedures, very difficult to standardize. The full complement of the signs and symptoms historically described as the "Charcot's triad" [1] or the "Reynolds' pentad" [2] are infrequent and, as such, do not really assist the clinician with planning management strategies. Few different consensus conference and severity score grading systems have been published from expert panels in recent years with consequent comments and criticisms [3-14]. Recently an International Consensus meeting held in Tokyo established evidence-based criteria for the diagnosis, severity assessment and treatment of acute cholecystitis (Tokyo guidelines). The Tokyo guidelines is a fine methodologically and scientifically correct study which defines the diagnostic and therapeutic approach to the acute biliary infections. Although many different diagnostic and treatment methodologies have been developed in recent years, none of them have been assessed scientifically to become a standard method in the management of acute biliary infections and, more specifically, acute cholecystitis. The Tokyo extraordinary expert panel, by a meticulous review of English-language literature, demonstrated that a structured diagnostic and severity scoring system for acute biliary infections is not available, and consequently tried to overcome this scientific gap.

The Tokyo guidelines offer a systematic overview and revision of the pathophysiological, clinic and diagnostic approach to the biliary infections. Based on this exhaustive overview these guidelines give also specific therapeutic indications about operative and conservative management.

The diagnosis is the starting point of the treatment of any kind of pathology and of acute cholecystitis as well. Prompt and timely diagnosis should allow early treatment and lower morbidity and mortality. The Tokyo guidelines proposed a staging system based upon the evaluation of local signs of inflammation (Murphy's sign and RUQ mass/pain/tenderness), systemic signs (fever, elevated CRP with values of 3 mg/dl or more and abnormal WBC count) and imaging findings characteristic of acute cholecystitis. Similar diagnostic criteria are reported from other recent studies [4,14].

As far as diagnosis and treatment of acute cholecystitis is concerned, the peculiarity of the Tokyo guidelines is the division of the disease in mild, moderate and severe [6,7]. No previous study examined the optimal treatment of acute cholecystitis on the basis of an organ-related severity score index. In the Tokyo consensus meeting the need for surgical treatment according to the grade of severity was suggested and discussed [7]. Subsequent studies analyzed the impact of the Tokyo guidelines on the management of patients with acute cholecystitis, stressing the attention on their impact on surgical outcomes. Even if the expert panel of that consensus made an extraordinary scientific work, no benefits have been demonstrated by applying those guidelines, except a decrease of mean length of hospital stay [8].

Acute cholecystitis could present with a picture ranging from mild, self limiting, to a potentially life threatening illness [6]. However the severity of inflammation and its life threatening potential is also strongly determined by the general condition of the patient, and the surgical treatment is often dictated more by the general conditions of the patient than by the grade of inflammation/infection of the gallbladder.

Actually no randomized controlled trials have examined the optimal surgical treatment for acute cholecystitis according to its severity grade and the panel at the Tokyo consensus meeting included patients with organ/systemic dysfunctions in the "grade III" of the guidelines, with the suggestion that these patients should receive delayed cholecystectomy after urgent drainage. Early gallbladder drainage is suggested also in grade II patients, with local severe inflammation, however a later systematic review of 53 papers about cholecystostomy as an option in acute cholecystitis found no evidence to support the recommendation of percutaneous drainage rather than straight early emergency cholecystectomy even in critically ill patients, and stated that it is not possible to make decisive recommendations about it. From their data, actually, cholecystectomy seems to be a better option than early drainage, for treating acute cholecystitis in the elderly and/or critically ill population [15].

Borzellino et al., in a recent review of prospective and retrospective series did not show an increase in local postoperative complications in laparoscopically treated severe (gangrenous and empyematous) acute cholecystitis but did not address the issue of timing of intervention in this subset of patients [16]. Once established the need for surgery, an accurate evaluation of the general condition of the patients should allow to exclude the surgical option in patients at high risk to not overcome the operation. For this subgroup of patients different options should be evaluated (e.g. percutaneous cholecystostomy) [17-20]. Patients whom general conditions allow to safely face surgery, acute cholecystitis should be operated by laparoscopy early after the beginning of symptoms [4,21-23]. In our opinion further investigations and studies should be undertaken in order to identify a more practical patient-related operative guidelines to treat acute cholecystitis and the issue of a scoring system that can be related to the clinical and therapeutic decision making is largely unresolved.
